# Adjacent habitat type affects the movement of predators suppressing soybean aphids

**DOI:** 10.1371/journal.pone.0218522

**Published:** 2019-06-18

**Authors:** Kandanpita Galaddalage Lahiru Ishan Samaranayake, Alejandro Carlos Costamagna

**Affiliations:** Department of Entomology, University of Manitoba, Winnipeg, Manitoba, Canada; University of Saskatchewan College of Agriculture and Bioresources, CANADA

## Abstract

Landscape complexity influences soybean aphid suppression by generalist predators in North America, but the role of adjacent habitats as sources of these predators has not been studied directly. We quantified movement of aphidophagous predators between soybean and five adjacent habitats common in Manitoba using bi-directional Malaise traps. To test the contribution of predators from neighboring habitats to soybean aphid suppression, we performed experimental manipulations in adjacent soybean and alfalfa fields and monitored the movement of sevenspotted lady beetles, *Coccinella septempunctata*, using mark-release-recapture experiments. The identity of adjacent habitats affected the net movement of predators into soybean. The most abundant predators were hover flies (Diptera: Syrphidae), moving from woodlands to soybean. Similar (but non-significant) trends were found for lady beetles, minute pirate bugs, and green and brown lacewings. There was also a net movement of hover flies and green lacewings from soybean to canola. Lady beetles showed higher bidirectional movement in alfalfa and wheat borders than in woodland and canola borders in a high lady beetle abundance year. Soybean aphid populations in predator exclusion cages were 21- to 122- fold higher than populations exposed to predators, both in alfalfa and soybean fields. Aerial predators provide similar levels of aphid suppression as aerial and epigeal predators combined. Mark-release-recapture experiments showed high dispersal of *C*. *septempunctata* between soybean and alfalfa, with a net movement towards alfalfa, probably due to the lack of aphids in soybean. These results demonstrate that predator assemblages from both soybeans and alfalfa can suppress soybean aphids. Our findings indicate that the type of adjacent habitat and predator identity affect the directionality of predator movement into soybean. This study suggests that information on predator movement can be used to design the distribution of crops and natural habitats in agricultural landscapes that maximize pest control services.

## Introduction

Most natural enemies change habitats during part of their life cycle to obtain food, mates, and reproductive sites [1], and associated pest control services depend largely on the movement of these predators through multiple habitats in agricultural landscapes [2,3]. However, the directionality of natural enemy movement (i.e. net immigration or emigration from a field) across habitat boundaries in agroecosystems has been quantified in relatively few studies. Duelli, Studer [4] found net emigration of lady beetles (Coleoptera: Coccinellidae) from corn into adjacent barley and wheat fields, using directional sticky cards in northwestern Switzerland. The same authors showed net immigration of carabid beetles into corn from wheat and barley borders, using directional pitfall traps [4]. Macfadyen and Muller [5] and Macfadyen, Hopkinson [6] conducted studies using bi-directional Malaise traps, and found differences in insect community composition (i.e. predators, parasitoids and herbivores) and in movement patterns of insects in canola and cereals (wheat / barley) associated with different adjacent habitats in Australia. Zumoffen, Signorini [7], also using bi-directional Malaise traps, found a net movement of aphid parasitoids (mainly Braconidae) from borders with natural vegetation towards wheat and alfalfa crops in Argentina. Marking methods suggest that dispersal of lady beetles within and between crops is affected by the number of aphids in the habitats studied [8,9], the crop type and phenology [10] and the lady beetle species [11]. Altogether, this previous research indicates the need for system-specific studies to quantify the movement of natural enemies between crops and other habitats to assess associated pest control services.

The soybean aphid, *Aphis glycines* Matsumura (Hemiptera: Aphididae), is an important pest that when abundant can reduce soybean yield significantly in North America [12–14]. Several studies have shown that aphidophagous predators, including different species of lady beetles (Coleoptera: Coccinellidae), minute pirate bugs (Hemiptera: Anthocoridae), damsel bugs (Hemiptera: Nabidae), larvae of hover flies (Diptera: Syrphidae), and lacewings (Neuroptera: Chrysopidae and Hemerobidae) suppress soybean aphid populations in North America [12,15–18]. Landscape complexity is associated with increased predator abundance and efficacy on soybean aphid suppression in some studies [19–21], but not in others [22]. Moreover, in a previous study we showed that predator movement rates explain patterns of soybean aphid suppression in soybean fields in Manitoba [23]. These studies suggest the need to study the role that landscape habitats (i.e. habitats and crop fields surrounding the focal field studied in the agricultural landscape) play in contributing predators to soybean (e.g. [24]).

Alfalfa (*Medicago sativa* L.) acts as a reservoir of many insect natural enemies in agricultural landscapes [25–27]. As a perennial crop, alfalfa harbours several aphid species throughout the growing season and natural enemies show numerical responses to aphid densities in alfalfa (e.g. [26]). In Australia, Costamagna, Venables [28] found that suppression of the melon aphid, *Aphis gossypii* Glover (Hemiptera: Aphididae), was positively associated with higher proportion of alfalfa in the landscape, suggesting that alfalfa acts as a source of aphidophagous predators. In Manitoba, several species of aphidophagous lady beetles, green lacewings, damsel bugs, and minute pirate bugs are commonly found in alfalfa [29]. The potential of the predator assemblage of alfalfa to suppress soybean aphid populations has not previously been tested.

Here, we determine the directionality of movement (i.e. immigration and emigration) of aphidophagous predators between soybean and the most common adjacent habitats in Manitoba, using bi-directional Malaise traps. In addition, we evaluate the potential of predator assemblages present in alfalfa fields to suppress soybean aphids in adjacent soybean fields using cage manipulations. Finally, we quantify the movement pattern of the sevenspotted lady beetle, *Coccinella septempunctata* L. (Coleoptera: Coccinellidae), within and between soybean and alfalfa fields, to demonstrate the potential of coccinellids to disperse to adjacent habitats and suppress aphids.

## Materials and methods

The study was carried out in private commercial soybean and alfalfa fields and prior authorization by field owners were obtained by phone or in person visiting their houses (often located by the fields where worked was performed). We also work at the experimental stations of the University of Manitoba (Carman and Glenlea) and were authorized by the field station managers; specific permits were not required for University students and staff to work on these stations.

### Sampling movement of predators between soybean and adjacent habitats

Patterns of predator movement between soybean and adjacent fields were studied in 12 (2013) and 15 (2014) fields in 12 localities in Manitoba: Altona, Arnes, Carman, Elm Creek, Emerson, Gimli, Glenlea, La Broquerie, Letellier, Morris, Rosewood, and Warren. In each focal soybean field, at least one type of adjacent habitat was sampled representing the most common crop and non-crop borders in Manitoba. A total of 30 field borders were studied including alfalfa (n = 7), canola (*Brassica napus* L.; n = 7), wheat (*Triticum* spp. L.; n = 3), border-grass (strips of vegetation around fields with a mixture of grass, broad leaf weeds, and wetland plants; n = 2) and woodland (natural and semi-natural vegetation dominated by trees and shrubs; n = 11). Townes style bi-directional Malaise traps (dimensions: 190 cm height at front, 160 cm length and 110 cm height at back; Sante Traps, Lexington KY, US) were established in each soybean field border to measure immigration and emigration of predators. Fifteen traps were established in field borders in 2013 (one trap was excluded from analysis due to deer damage) and 16 traps in 2014. In 2014, eight additional traps were deployed in eight soybean fields (100 m from the field border) as controls to compare movement patterns within and between habitats. The two collection bottles of each bi-directional Malaise trap were filled with 70% ethanol (~375 ml) and were changed weekly from 22^nd^ July to 16^th^ August in 2013 (3 weeks) and from 28^th^ July to 28^th^ August in 2014 (4 weeks). Adjacent canola fields were flowering for 2–3 weeks during our study. Captures during the two initial weeks of both years of study were previously used to relate overall levels of predator movement between soybean and neighboring fields with aphid suppression in soybean [23]. Here we expanded this dataset by adding three sampling weeks, allowing us to test for the effect of neighboring habitat type on rates of predator immigration versus emigration to soybeans (all combined in the analysis of the previous subset of the data). Insects were stored in 70% ethanol until they were identified. Aphidophagous predators were counted and identified to family and species when possible, using taxonomic keys. Hover fly and a few green lacewing species identities were confirmed by taxonomists at the Canadian National Collection of Insects, Arachnids and Nematodes. Voucher specimens were deposited in the Wallis-Roughley Museum of Entomology, University of Manitoba, Canada. Soybean aphid abundance was assessed by visually counting aphids on 20 plants per field during the three initial weeks of sampling each year [23], no assessments of insect abundance were conducted in adjacent crops.

### Potential of assemblages of predators in alfalfa to suppress soybean aphid

To test predation on soybean aphids, we used potted soybean plants (*Glycine max* (L.) Merr., Fabaceae, variety OAC Prudence, Shanawan Farms, Domain, MB, Canada) with sentinel aphids, following the methods described in [23]. Plants were grown in square plastic pots (9 cm x 9 cm x 18 cm high) using equal parts of peat mix (Sunshine Mix#4, Sun Gro Horticulture Canada Ltd. Seba Beach, Alberta, Canada) and sand, in greenhouse conditions (16:8 h L: D; 23°–27°C, and 60–75% RH). Plants used for the experiments were at the V3 –V4 vegetative stage [30].

The experiment was conducted between July 16 and August 2, 2012 at four separate locations in Manitoba that included experimental plots at the Carman and Glenlea experimental stations of the University of Manitoba, and production fields located in the Rural Municipalities of La Broquerie and Giroux. In each location, one soybean and one adjacent alfalfa field were selected to test the impact of different predator guilds on soybean aphid populations. Adult or near-adult soybean aphids from a laboratory colony were manually transferred to two potted soybean plants (7 aphids per plant) using a fine paint brush. Three predator manipulation treatments were set up: 1) exposure to all predators (epigeal + aerial predator treatment), 2) exposure to aerial predators only (i.e. aerial predator treatment) and 3) protected from all predators (i.e. predator exclusion control). We included an aerial predator treatment because 1) this is the group of predators that is likely to move between adjacent habitats, and 2) previous studies showed different levels of aphid suppression by aerial and epigeal predators [31]. The epigeal + aerial predator treatment consisted of potted soybean plants buried to ground level and exposed to ambient levels of predators. Neighboring soybean plants were removed to prevent aphid movement between plants in all three treatments [32]. The aerial predator treatment was set up by burying potted plants only half way into the ground. The side of the pot left above ground level (approximately 10 cm) was coated with Tanglefoot (The Tanglefoot Company, Grand Rapids, Michigan) to act as a barrier for epigeal predators. The predator exclusion treatment consisted of partially buried potted plants (as in the aerial predator treatment) covered by sleeves of fine mesh (white no-see-um white netting with 0.24 mm^2^ openings). The sleeves were supported by cylindrical tomato cage frames (1 m tall x 0.4 m diameter) and buried in the soil, following the design described in Samaranayake and Costamagna [23]. The mesh was tied at the top of the cage and was pulled down for weekly counts. Each treatment was replicated five times in each soybean and alfalfa field studied. Replicates were separated by 1–2 m and were 10 m from the field border.

Potential effects of aphid or host plant species on levels of aphid suppression observed in alfalfa were assessed by repeating the design described above using pea aphids (*Acyrthosiphon pisum* Harris, Hemiptera: Aphididae) on field alfalfa plants. The epigeal + aerial predator treatment consisted of 4–6 alfalfa stems that were cleared of all existing insects and re-infested with 10 pea aphids. We used a smaller number of pea aphids per plant than soybean aphids in an attempt to compensate for the larger size of pea aphids. The patch with manipulated alfalfa stems was marked with wire flags and separated from other alfalfa plants by clearing adjacent alfalfa stems. The aerial predator treatment consisted of similarly manipulated alfalfa stems that were protected from epigeal predators by a PVC ring (22 cm diameter x 17.5 cm tall, 9 mm wall), partially buried (5 cm), and secured into the ground with two camping stakes on opposite sides. The upper 5 cm of the inside and outside of the ring walls were coated with Tanglefoot. Finally, the predator exclusion treatment used the same cage design as for soybean aphids, but replacing the potted soybean plants by the alfalfa stems as described above. This design was replicated 5 times in each alfalfa field in each location. Soybean and pea aphids were counted once a week in each treatment, for a total of 180 experimental populations studied. In all predator exclusion and aerial predator treatments, we included one small pitfall traps (6 cm diameter x 7 cm height) to remove any epigeal predators that we may have missed during our initial inspections. No aphids were observed on the Tanglefoot barrier and only a handful were found in the pitfall traps in exclusion cages, suggesting that these were only minor mortality factors that did not bias our results.

Abundance of aphids and predators in each field used for cage experiments was assessed weekly during the experiment using sweep-net sampling and sticky traps. Sweep-net sampling consisted of 5 subsamples / field, each of 25 sweeps, conducted haphazardly at least 5 m from cage manipulations and 10 m from the field border, during the initial setup, first week, and second week of the experiment (n = 3 samples / field). Five yellow sticky traps (Pherocon Unbaited AM Yellow Sticky Traps) were deployed weekly (mean = 6.2 days / sticky trap sample, period varied due to logistical reasons) in each field, within 1–2 m from cage manipulations, during the cage experiment.

### Movement of marked predators between soybean and alfalfa

Two mark-release-recapture experiments were carried out in adjacent commercial soybean and hay alfalfa fields in Gimli, Manitoba (50°34'55.0"N, 97°00'36.9"W) from 10 to 12 July in 2013 and at the Ian N. Morrison Research Farm, in Carman, Manitoba (49°30'06.3"N, 98°01'34.9"W) from 23 to 25 July in 2014. Field sites were selected based on soybean and alfalfa having similar plant heights during the experimental period, and sites were only selected when there were no barriers between fields. Soybean fields had similar row spacing (50 cm) in both years. Adult sevenspotted lady beetles, *C*. *septempunctata*, were used for this study as they were the most abundant lady beetle species found in Manitoba [29]. Lady beetles were collected two days prior to each experiment by sweep-netting in alfalfa and wheat fields at the Glenlea Research Station of the University of Manitoba and were kept at 5°C. Lady beetle elytra were painted with one of six different combinations of patterns of spots and colours (light blue or yellow, Extratine, Decocolor Opaque Paint marker, Uchida of America Corporation), to identify the release points. After marking, lady beetles were transferred into ventilated containers (≤ 28 lady beetles / container; 4 containers / release point) and kept at 5°C for 24 h until release. Preliminary laboratory experiments confirmed that storage temperatures and marking procedure did not cause lady beetle mortality [33]. To reduce disturbance-induced dispersal, all marked lady beetles were released at 10:00 a.m. on 12 July 2013 and at 9:00 a.m. on 23 July 2014, when air temperatures were still cool [9]. Periodic inspections of the release points were conducted during the initial two hours after release, ensuring that lady beetles were leaving the containers and no predators were attacking them.

Three lady beetle release points in alfalfa and three release points in soybean were established 12 m from the soybean-alfalfa border. A previous mark-release-recapture study found that 30 m was the maximum recapture distance for *C*. *septempunctata* in alfalfa after 24 hours [9]. Therefore, release points were established 12 m from the soybean-alfalfa border along the three central transects (see below), separated by 4 m, in order to ensure lady beetles could move between fields within a day. A total of 654 and 600 marked lady beetles were released in 2013 and 2014, respectively. In each year, an equal number of marked lady beetles were released at each of the six release points. The sampling area consisted of a rectangular area that spanned both the alfalfa and the soybean fields. Seven transects separated by 4 m were laid out into each crop, perpendicular to the soybean-alfalfa field border. Sampling points along transects were established at 3 m intervals. Results from the 2013 experiment indicated that the maximum distance at which lady beetles were recaptured between crops exceeded the maximum distance sampled within each crop. To avoid bias in the comparisons, in the 2014 experiment, transect length was increased from 72 m (2013) to 102 m (increasing total recapture points from 168 to 238, respectively). Five sweeps were taken between two sampling points along transects and marked and unmarked lady beetles were counted and released immediately. The original release point for each of the recaptured beetles was determined by their mark. Sweep-net samples along transects were taken 2, 4, 6, 8, 24, 26, 28, 30, 32 and 48 hours after the release. In addition to the sweep-netting, 6 (2013) and 10 (2014) Townes style bi-directional Malaise traps were established at the border between alfalfa and soybean (4 m away from the two outer transect lines). Collection bottles were filled with soapy water (~350 ml) and replaced every 24 hours during the study period. Marked and unmarked lady beetles captured in the traps were identified and recorded separately for each trap. Sampling of field populations of aphids and aphidophagous predators was conducted outside the mark-release-recapture sampling area using standard sweep-net sampling (25 sweeps / sample, six samples / field). Aphidophagous predators and aphids captured in sweep-net samples were identified to family level and their numbers were recorded. During the sampling period, mean temperature was 16.9°C (range 5.8–26.9°C), precipitation was minimal (mean 1.72 mm, range 0–8.6 mm), and the wind was calm (mean speed 7.6 km / h, range 4.8–13.7 km / h).

### Data analysis

#### Captures of predators moving between soybean and adjacent habitats

All statistical analyses were conducted in R [34]. Linear mixed-effect models were used to test the effects of adjacent border (alfalfa, canola, border-grass, woodland, and wheat), sampling year (2013 and 2014) and directionality predator movement (i.e. immigration versus emigration to soybean) and their 2- and 3-way interactions on the number of predators captured on each side of the bi-directional Malaise trap. Since immigration and emigration were quantified in the same trap, direction of movement was nested within trap, which was modeled as a random effect. All aphidophagous predators combined (i.e. total aphidophagous predators) and totals per family, were used as response variables in separate models. Counts were averaged per bottle and per day to account for different number of weeks sampled each year and different sampling intervals that occurred in some weeks due to rain. Counts were log-transformed (log10 [counts + 1]) before analysis, to meet model assumptions. Stepwise backward selection was used to select the best final model by deleting non-significant interaction terms to improve model fit. Linear mixed-effect models were fit using the function “lme” in the library “nlme” in R [35]. The significance of interaction terms was tested using the ‘anova’ function on maximum likelihood estimates of model parameters to obtain *p*-values from likelihood ratio tests [36], and the level of improvement of the model was estimated using Akaike Information Criterion (AIC). Contrasts of least-squares means adjusted by the Tukey method for multiple comparisons were used to conduct pairwise comparisons between treatments within significant 2-way interaction terms, using the “lsmeans” package in R [37]. Either paired t-tests or paired Wilcoxon rank sum tests with continuity corrections were used to compare numbers moving towards the field interior with numbers moving towards the field margin in control bi-directional Malaise traps, and combined movement (i.e. average immigration and emigration) between control and border bi-directional Malaise traps.

#### Potential of assemblages of predators in alfalfa to suppress soybean aphid

To compare predation on soybean versus alfalfa fields, we analyzed the number of soybean aphids (10^th^ root-transformed to achieve normality and homocedasticity) after two weeks of manipulation with a split-plot ANOVA model, with crop as the whole-plot factor and predator manipulation as the sub-plot factor. To account for different initial numbers of aphids used in soybean aphid and pea aphid treatments in alfalfa fields, we calculated the per capita rate of increase of aphids (λ^T^) after two weeks of manipulation (aphid number after two weeks / initial aphid number; [38]). Despite efforts to remove resident aphids from alfalfa stems during setup, some spotted alfalfa aphids, *Therioaphis maculata* (Buckton) (Hemiptera: Aphididae), remained on the plants (10.3% of total aphids); only pea aphids were used for all statistical analysis. Predation on soybean aphids versus pea aphids was compared using a factorial design with aphid species, predator manipulation treatments and their interaction as fixed effects. In all analyses, location was included as a random blocking factor. To avoid pseudoreplication, the average of each predator manipulation treatment per field was analyzed. Predator manipulation treatments were compared with least square mean pairwise comparisons adjusted by the Tukey method for multiple comparisons. Aphid and predator abundances were averaged per field and sampling week for each sampling method and compared between crops using paired Wilcoxon rank sign tests, due heterocedasticity in the counts (n = 8 for sticky traps and n = 12 for sweep-net samples).

#### Movement of marked predators between soybean and alfalfa

The number of recaptured lady beetles in sweep-net samples was compared within and between crops with Kruskal-Wallis rank sum tests with pairwise comparisons adjusted by the Sequential Bonferroni method [39]. All samples that yielded zero lady beetles in equivalent positions in the soybean and the alfalfa fields were eliminated to simplify statistical analysis. One-way ANOVA was used to compare predator abundance in sweep-net samples between alfalfa and soybean within and between years. For all parametric tests, normality of the data and homogeneity of variance were visually checked using normal Q-Q plots and heteroscedasticity plots. Unless otherwise indicated, all reported values are mean ± SEM, and α = 0.05 was used to assess significant differences.

## Results

### Predators moving between soybean and adjacent habitats

A total of 25,460 aphidophagous predators (including adult stages of species that are predatory as juveniles) were captured moving between soybean and adjacent habitats using bi-directional Malaise traps; with an average of 13.18 ± 1.25 individuals / bottle / day (Table 1). The aphidophagous guild included six insect families and was dominated by Syrphidae (hover flies, 89.75% of total capture), followed by Anthocoridae (minute pirate bugs, 4.56%), Coccinellidae (lady beetles, 1.27%), Chrysopidae (green lacewings, 0.64%), Hemerobiidae (brown lacewings, 0.58%), and Nabidae (damsel bugs, 0.04%) (Table 1). *Toxomerus marginatus* (Say) (Diptera: Syrphidae) represented 94.55% of the aphidophagous hover flies. *Coccinella septempunctata* represented 59.77% of the lady beetles, followed by the thirteenspotted lady beetle, *H*. *tredecimpunctata* (23.56%), and the multicoloured Asian lady beetle, *Harmonia axyridis* (13.79%) (Table 1). *Chrysoperla carnea* (Stephens) represented 72.20% of the green lacewings (Table 1).

**Table 1:** Average number of predators (standardized per day) captured on 38 bi-directional Malaise traps (14 traps in 2013 and 24 in 2014) in Manitoba, Canada, for 3 weeks in 2013 and 4 weeks in 2014 (n = 276).

Order	Family	Species	Individuals / bottle / day^5^	% of total
Coleoptera	Coccinellidae^1^		(0.174)	(1.273)
		*Coccinella septempunctata*^3^ Linnaeus, 1758	0.104	0.765
		*Hippodamia tredecimpunctata*^3^ (Linnaeus, 1758)	0.041	0.300
		*Harmonia axyridis*^3^ (Pallas, 1773)	0.024	0.174
		*Hippodamia variegata*^3^ (Goeze, 1777)	0.002	0.015
		*Psyllobora vigintimaculata*^3^ (Say, 1824)	0.001	0.008
		*Chilocorus* sp.^3^	0.001	0.007
		*Hyperaspis conviva* ^3^Casey, 1924	0.001	0.004
Diptera	Syrphidae^1^		(12.525)	(92.077)
		Aphidophagous hover flies^2^	(12.209)	(89.752)
		*Toxomerus marginatus*^3^ (Say, 1823)	11.543	84.857
		*Eupeodes latifasciatus*^3^ (Macquart, 1829)	0.264	1.937
		*Eupeodes volucris*^3^ Osten Sacken, 1877	0.251	1.844
		*Toxomerus geminatus*^4^ (Say, 1823)	0.163	1.197
		*Sphaerophoria contigua*^4^ Macquart, 1847	0.073	0.539
		*Sphaerophoria philanthus*^3^ Meigen	0.052	0.384
		*Platycheirus hyperboreus*^3^ (Staeger, 1845)	0.042	0.312
		*Platycheirus nearcticus*^4^ Vockeroth, 1986	0.021	0.155
		*Parhelophilus laetus*^4^ (Loew, 1963)	0.018	0.001
		*Syrphus rectus*^3^ Osten Sacken, 1875	0.018	0.133
		*Syritta pipiens*^4^ (Linnaeus, 1758)	0.015	0.110
		*Eumerus strigatus*^4^ (Fallen, 1817)	0.014	0.102
		*Eupeodes americanus*^3^ (Wiedemann, 1830)	0.013	0.099
		*Allograpta obliqua*^3^ (Say, 1823)	0.010	0.076
		*Platycheirus immarginatus*^3^ (Zetterstedt, 1849)	0.009	0.068
		*Eupeodes (Lapposyrphus) lapponicus*^4^ (Zetterstedt, 1838)	0.005	0.038
		*Chrysotoxum derivatum*^4^ Walker, 1849	0.004	0.026
		*Syrphus ribesii*^3^ (Linnaeus, 1758)	0.002	0.015
		*Melanostoma mellinum*^3^ (Linnaeus, 1758)	0.002	0.011
		*Ocyptamus fuscipennis*^3^ (Macquart, 1834)	0.001	0.008
		*Paragus haemorrhous*^3^ Meigen, 1822	0.001	0.008
		*Neocnemodon* sp.^4^	0.001	0.007
		*Platycheirus granditarsis*^4^ (Forster, 1771)	0.001	0.004
		*Ferdinandea buccata*^4^ (Loew, 1863)	0.001	0.004
		*Lejops (Eurimyia) lineatus*^4^ (Fabricius, 1787)	0.001	0.004
		*Helophilus fasciatus*^4^ Walker, 1849	0.001	0.004
Hemiptera	Anthocoridae^1^	*Orius insidiosus*^3^ (Say, 1832)	0.621	4.564
	Nabidae^1^^,^ ^3^		0.005	0.038
Neuroptera	Chrysopidae^1^		(0.199)	(1.465)
		Aphidophagous green lacewings^2^	(0.087)	(0.641)
		*Chrysoperla carnea*^3^ (Stephens, 1836)	0.063	0.463
		*Chrysopa* sp.^3^	0.024	0.178
		*Chrysoperla* spp.^6^	0.111	0.817
		*Ceraeochrysa lineaticornis* ^4^ (Fitch, 1855)	0.001	0.008
	Hemerobiidae^1^^,^ ^3^		0.079	0.582
		**Total (all predators)**	**13.603**	**100**

^1^ Higher taxonomic levels of aphidophagous predators used for analysis of immigration and emigration.

^2^ Abundances of aphidophagous taxa in Syrphidae and Chrysopidae families were used for analysis of immigration and emigration.

^3^ Aphidophagous taxon.

^4^ Non-aphidophagous taxon (not used for statistical analysis).

^5^ Average number of individuals adjusted to 1-day intervals, as 8-day intervals due to rain occurred in some fields.

^6^ Not included in multiple regression models as it was not possible to determine aphidophagy at this taxonomic level.

Values between parentheses are not included in total numbers at the bottom of the table.

**Fig 1:**
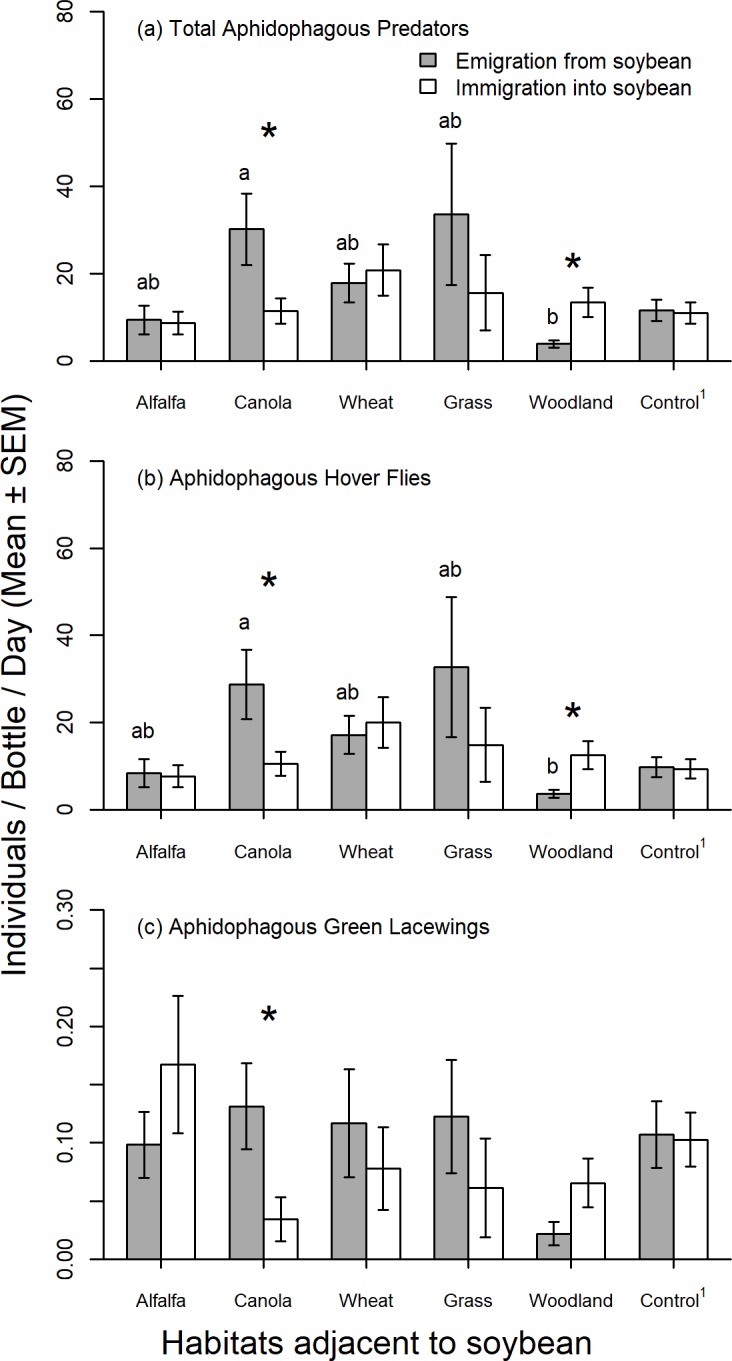
**Average daily emigration and immigration of (a) total aphidophagous predators, (b) aphidophagous hover flies, and (c) aphidophagous green lacewings between soybean and different adjacent habitats, combining two years of sampling (7 weeks in total).** Sampling consisted of bi-directional Malaise traps established on five adjacent habitats: soybean-alfalfa (n = 24 bottles), soybean-canola (n = 25), soybean-grass (n = 7), soybean-woodland (n = 39), soybean-wheat (n = 11) and control (soybean fields, 100 m from the field border, n = 32). Significant differences (*p* < 0.05) between emigration and immigration (or captures towards the field interior and field margin in controls) are indicated with *, and for emigration levels among field borders with different lower case letters; no significant differences were observed in immigration levels. ^1^ Control bi-directional Malaise traps were established in a subset of fields in 2014 (n = 8 fields).

**Fig 2:**
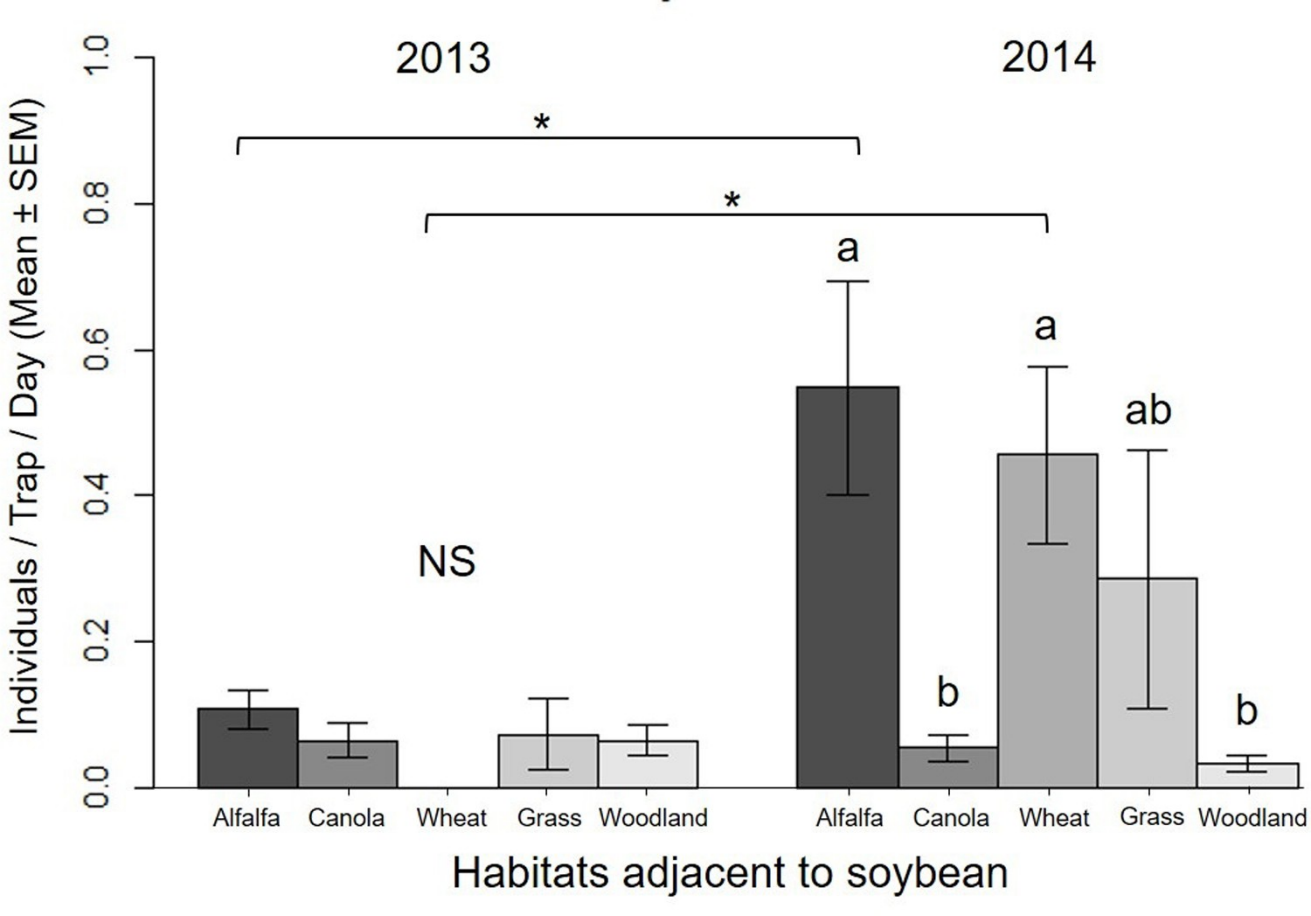
Average daily captures of lady beetles between soybean and different adjacent habitats combining totals from both sides of bi-directional Malaise traps, during 2013 and 2014. Different lower case letters indicate significant difference in captures of lady beetles among adjacent habitats (multiple comparisons of least-square means adjusted by Tukey, *p* < 0.05; NS = not significant) significant differences between years within habitats are indicated by * (*p* < 0.05).

Overall captures of predators were higher in 2014 (16.76 ± 1.68 individuals / bottle / day, n = 192) than in 2013 (4.98 ± 1.08, n = 84; Table 2). There was no difference between overall emigration and immigration of total predators between soybean and adjacent habitats (14.72 ± 2.58 versus 12.78 ± 1.73 individuals / trap / day, respectively; n = 106), but movement direction varied among field borders (significant border x migration; Table 2). Emigration rates from soybean to canola were higher than to woodland and emigration to all other borders were intermediate (Fig 1A). There was no difference in immigration levels to soybean among field borders (Fig 1A). We found a net emigration to canola and a net immigration from woodland (Fig 1A), but we did not observed directionality of total predator movement in other field borders. Aphidophagous hover flies were the numerically dominant predator group and consequently they show the same pattern as all predators combined, with higher captures in 2014 (18.41 ± 2.29 individuals / bottle / day, n = 128) than in 2013 (4.73 ± 1.07, n = 84; Table 2); higher emigration to canola than to woodland; higher net emigration to canola; and higher net immigration from woodland (Fig 1B). Aphidophagous green lacewing captures were similar in both years, but the directionality of movement differed among field borders due to higher net emigration to adjacent canola (Table 2 and Fig 1C). Overall, lady beetle immigration was higher than emigration (0.19 ± 0.04 versus 0.12 ± 0.03 individuals / trap / day, respectively; n = 106), and was higher in 2014 than in 2013 (0.20 ± 0.04, n = 128, versus 0.07 ± 0.01, n = 84, respectively), but varied among adjacent habitats to soybean (Table 2 and Fig 2). Captures of lady beetles were higher in alfalfa and wheat borders in 2014 than in 2013 (Fig 2). In 2014, captures of lady beetles were higher in alfalfa and wheat borders compared to canola and woodland, and were intermediate in grass border (Fig 2). There were no differences in the numbers of lady beetles captured among habitats adjacent to soybean in 2013 (Fig 2).

**Table 2:** Results of linear mixed-effects models for total predators, hover flies, green lacewings, and lady beetles captured in bi-directional Malaise traps, with border (alfalfa, canola, border-grass, woodland, and wheat), migration (immigration to vs emigration from soybean), year (2013 and 2014) and their interactions.

Factor	Total predators				Hover flies				Green lacewings				Lady beetles			
	*df*_num_	*df*_den_	*F*	*P*	*df*_num_	*df*_den_	*F*	*p*	*df*_num_	*df*_den_	*F*	*p*	*df*_num_	*df*_den_	*F*	*p*
Border	4	24	1.52	0.220	4	24	1.49	0.235	4	24	1.35	0.280	4	20	7.76	0.001
Year	1	24	12.48	0.002	1	24	10.37	0.004	1	24	1.36	0.254	1	20	11.19	0.003
Migration	1	25	0.22	0.640	1	25	0.05	0.829	1	25	0.01	0.943	1	29	6.82	0.014
Border × Migration	4	25	6.95	0.001	4	25	5.67	0.002	4	25	2.88	0.043	-	-	-	-
Border × Year	-	-	-	-	-	-	-	-	-	-	-	-	4	20	6.59	0.002

Interactions between Year × Migration and Border × Year × Migration were not significant for any of the four predator variables presented here.

Captures of minute pirate bugs were higher in 2014 (0.72 ± 0.11 individuals / bottle / day, n = 128) than in 2013 (0.03 ± 0.01, n = 84; *F*_1, 20_ = 27.95, *p* < 0.001), but did not differ by borders (*F*_4, 20_ = 2.47, *p* = 0.08), or movement directions (*F*_1, 29_ = 0.13, *p* = 0.71). Similarly, captures of brown lacewings were higher in 2014 (0.11 ± 0.02 individuals / bottle / day, n = 128) than in 2013 (0.03 ± 0.01, n = 84; *F*_1, 24_ = 7.97, *p* = 0.009), but did not differ by borders (*F*_4, 24_ = 1.43, *p* = 0.25) or movement directions (*F*_1, 29_ = 2.14, *p* = 0.15). Captures of damsel bugs did not differ by borders (*F*_2, 22_ = 0.14, *p* = 0.87), movement directions (*F*_1, 22_ = 1.22, *p* = 0.28), or years (*F*_1, 22_ = 2.58, *p* = 0.12). Despite the lack of differences resulting from low captures and high variability in the field samples, there was an overall trend of higher immigration from woodland into soybean for most groups sampled (S1 Fig).

In control traps, located 100 m from the field border, there was no difference in the number of predators captured between the two sides of the trap when all predators were combined (Fig 1A), and when each predator group was compared separately (separate paired t-tests per group, all *p* > 0.05), except for brown lacewings (*t* = 3.32, df = 31, *p* = 0.0023). Combining captures in both sides of the trap revealed similar quantities of all predators combined in control traps (11.3 ± 2.3 individuals / bottle / day) and in border traps (13.3 ± 3.0; *t* = 0.88, df = 31, *p* = 0.39). Separate predator groups also showed no differences in overall movement between border and control (lady beetles: control 0.26 ± 0.07 individuals / bottle / day, border 0.26 ± 0.07, t = -1.13, df = 31, *p* = 0.89; hover flies: 9.63 ± 2.07, 12.1 ± 2.9, t = -1.13, df = 31, *p* = 0.26; green lacewings: 0.1 ± 0.02, 0.08 ± 0.02, t = -1.12, df = 31, *p* = 0.26; brown lacewings: 0.1 ± 0.03, 0.08 ± 0.01, Wilcoxon test, *p* = 0.55; minute pirate bugs: 1.2 ± 0.25, 0.8 ± 0.18, t = -2.57, df = 31, *p* = 0.08; damsel bugs: 0.002 ± 0.002, 0.002 ± 0.002, t = 0, df = 31, *p* = 1.0). Soybean aphid populations were very low in the fields studied during both years (2013: 0.16 ± 0.06 aphids / plant, n = 720 plants, and 2014: 4.35 ± 0.74 aphids / plant, n = 900 plants; [23]).

### Potential of the assemblage of predators in alfalfa to suppress soybean aphid

**Fig 3:**
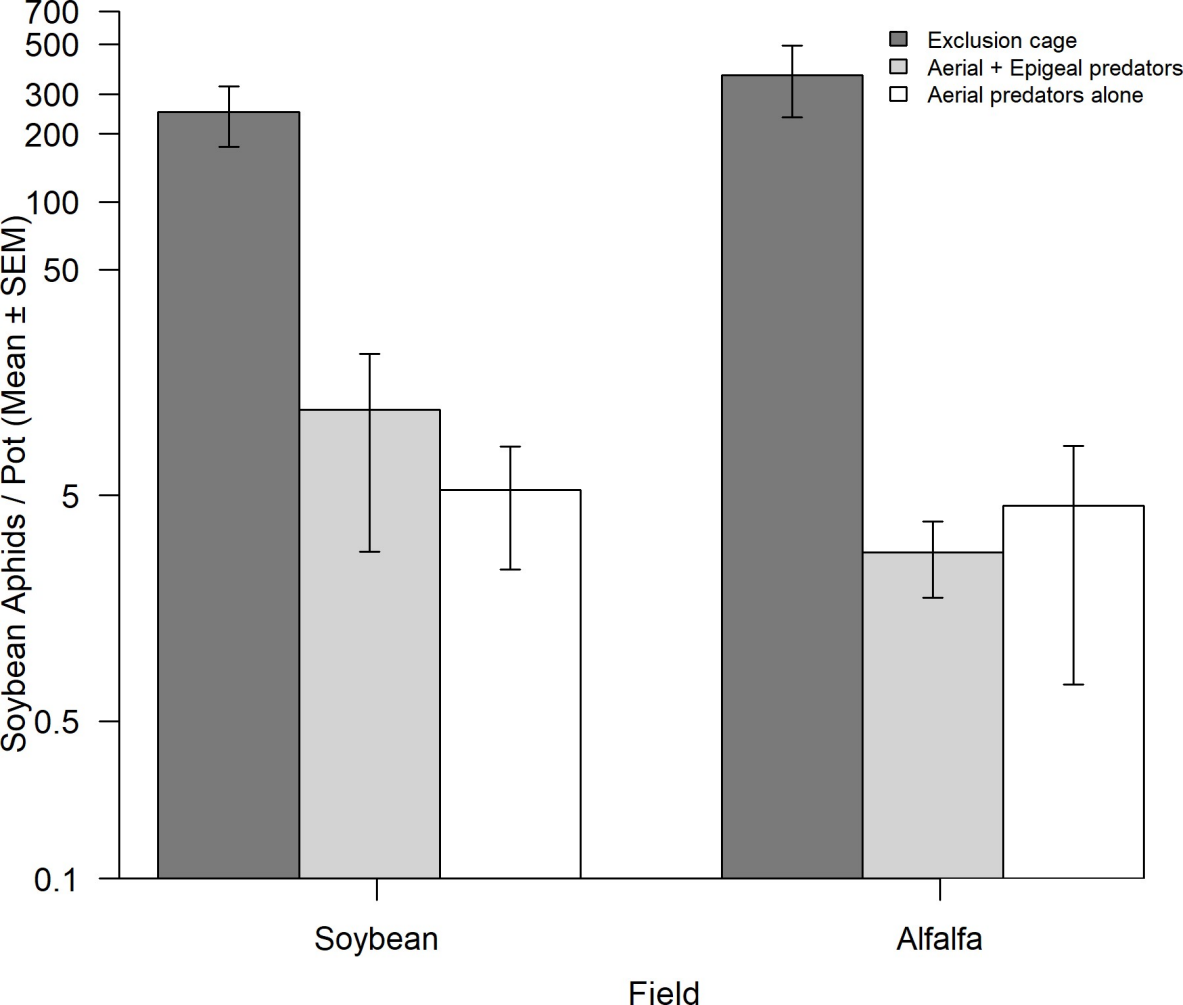
Final *A*. *glycines* numbers (mean ± SE) after two weeks subject to different types of predation in alfalfa and soybean fields in Manitoba. Aphid numbers were not significantly affected by crop, but were significantly reduced by the action of aerial predators alone and aerial + epigeal predators (see text for details).

Final soybean aphid numbers did not differ in alfalfa and soybean fields (crop: *F*_1, 3_ = 0.302, *P* = 0.621; crop x predator manipulation: *F*_1, 12_ = 1.008, *P* = 0.394), but were between 21- to 122- fold higher when protected from predators (predator manipulation: *F*_2, 12_ = 69.239, *P* < 0.0001, Fig 3). Aerial predators alone and aerial + epigeal predators resulted in lower numbers of aphids than the predator exclusion control (*t*_12_ = 9.72, *P* < 0.0001, and *t*_12_ = 10.61, *P* < 0.0001, respectively), but did not differ between them (*t*_12_ = 0.89, *P* = 0.6579) (Fig 3). We found a similar result when we compared predation on soybean aphids versus pea aphids in alfalfa fields. Per capita rates of increase of pea aphids and soybean aphids did not differ (aphid species: *F*_1, 15_ = 0.773, *P* = 0.393; aphid species x predator manipulation: *F*_2, 15_ = 0.765, *P* = 0.483), but predation had strong negative effects on both species (predator manipulation: *F*_2, 15_ = 85.400, *P* < 0.0001). Aphids in predator exclusion treatments (pea aphids: 20.9 ± 3.1 aphids / aphid; soybean aphids: 17.4 ± 5.4 aphids / aphid) had 19- to 20- fold higher per capita rates of increase than when exposed to aerial predators (pea aphids: 0.69 ± 0.33 aphids / aphid; soybean aphids: 0.37 ± 0.21 aphids / aphid; *t*_1,15_ = 11.6, *P* < 0.0001) or to aerial + epigeal predators (pea aphids: 0.42 ± 0.18 aphids / aphid; soybean aphids: 0.86 ± 0.66 aphids / aphid; *t*_1,15_ = 11.1, *P* < 0.0001). Per capita rates of increase did not differ between treatments exposed to predators (*t*_1,15_ = 0.49, *P* = 0.88).

A total of 19,734 aphids were collected with sweep nets in alfalfa (34.8% pea aphid, 63.0% spotted alfalfa aphid, 2.2% unidentified aphid species), whereas no soybean aphid colonies were found in soybean fields (only 21 alate aphids from other species were collected in soybean; Wilcoxon test, *P* < 0.0005, Table 3). Significantly higher numbers of predators were collected in sweep-net samples in alfalfa than in soybean, including *Orius insidiosus* (Hemiptera: Anthocoridae, 1861 versus 245 individuals, respectively), several species of Nabidae (837 versus 189), Aranea (272 versus 64), Coccinellidae (145 versus 5), Chrysopidae (85 versus 36), and Syrphidae (74 versus 10) (Wilcoxon tests, *P* < 0.05, Table 3). Staphilinidae (77) and Hemerobidae (16) did not differ between crops (Table 3). This trend did not hold for predators captured on sticky traps, where only Coccinellidae were significantly more abundant in alfalfa than soybeans (Table 3). Only *O*. *insidiosus* showed significantly more abundance in soybean than in alfalfa (264 versus 131, *P* = 0.013, Table 3), probably due to the absence of nymphs in sticky trap samples. Minute pirate bugs were the most frequently collected predator in both collection techniques, but otherwise the most frequently collected varied with the technique. Damsel bugs were the second most frequently detected predator using sweep net sampling, yet were not detected by the sticky traps.

**Table 3:** Average number of predators (± SE) captured on yellow sticky traps (n = 8 samples, 4 fields x 2 crops x 2 weeks of sampling) and sweep nets (n = 12 samples, 4 fields x 2 crops x 3 sampling dates) in paired alfalfa and soybean fields in Manitoba, Canada, in 2012.

Sampling	Taxon	Common name	Total collected	Alfalfa	Soybean	*p* value*
				Mean ± SE	Mean ± SE	
Sticky traps	Anthocoridae	Minute pirate bugs	395	1.72 ± 0.44	3.03 ± 0.44	**0.0156**
	Chrysopidae	Green lacewings	332	2.64 ± 1.03	1.65 ± 0.36	0.5469
	Syrphidae	Hover flies	307	1.46 ± 0.34	2.46 ± 0.66	0.0920
	Coccinellidae	Lady beetles	297	3.44 ± 1.07	0.75 ± 0.16	**0.0078**
	Order: Aranea	Spiders	210	0.87 ± 0.17	1.69 ± 0.54	0.2070
	Staphilinidae	Rove beetles	77	0.49 ± 0.20	0.38 ± 0.09	1.0000
	Hemerobiidae	Brown lacewings	16	0.07 ± 0.06	0.11 ± 0.09	0.6845
						
Sweep nets	Aphididae	Aphids	19734	359.83 ± 97.44	0.35 ± 0.12	**0.0005**
	Anthocoridae	Minute pirate bugs	2106	32.80 ± 10.73	4.08 ± 0.81	**0.0005**
	Nabidae	Damsel bugs	1026	15.36 ± 2.38	3.15 ± 0.67	**0.0025**
	Order: Aranea	Spiders	336	5.20 ± 1.69	1.07 ± 0.19	**0.0342**
	Coccinellidae	Lady beetles	150	2.81 ± 1.01	0.08 ± 0.04	**0.0058**
	Chrysopidae	Green lacewings	121	1.48 ± 0.38	0.60 ± 0.21	**0.0438**
	Syrphidae	Hover flies	84	1.24 ± 0.35	0.17 ± 0.06	**0.0165**
	Staphilinidae	Rove beetles	35	0.70 ± 0.66	0.02 ± 0.02	0.3711
	Hemerobiidae	Brown lacewings	13	0.19 ± 0.11	0.05 ± 0.03	0.2809

* Wilcoxon signed rank test paired

### Movement of marked predators between soybean and alfalfa

**Fig 4:**
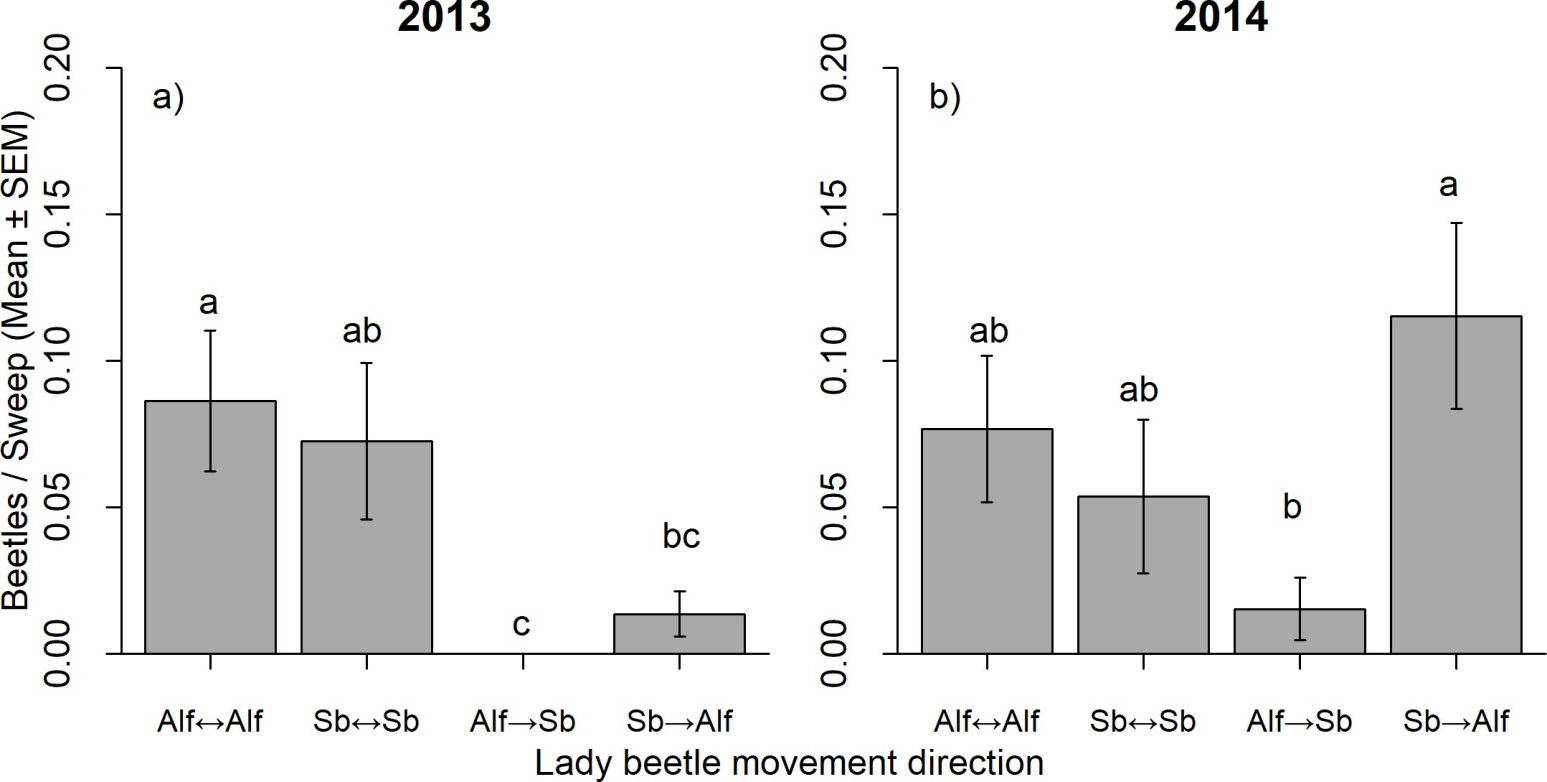
**Average captures of marked lady beetles, *C*. *septempunctata*, within and between soybean and alfalfa fields in 2013 (a) and 2014 (b).** Lower case letters represent significant differences (*p* < 0.05) between captures of marked lady beetles within and between fields (overall Kruskal-Wallis test followed by Kruskal-Wallis pairwise comparisons adjusted by Sequential Bonferroni). When one of the treatments was zero, a one sample t-test with H_o_ Mean = 0 was used. Movement directions: Alf↔Alf: alfalfa to alfalfa, Sb↔Sb: soybean to soybean, Alf→Sb: alfalfa to soybean, Sb→Alf: soybean to alfalfa.

Thirty-eight (5.8% of released individuals) and 34 (5.7%) sevenspotted lady beetles were recaptured in 2013 and 2014, respectively. Movement of lady beetles differed within and between crops in 2013 (Kruskal-Wallis χ^2^ = 17.20, df = 3, *p* < 0.001) and 2014 (Kruskal-Wallis χ^2^ = 9.70, df = 3, *p* < 0.05). In 2013, movement from soybean to alfalfa was approximately 20% of the movement observed within crops (Fig 4A). No individuals released in alfalfa were captured in soybean (Fig 4A). In 2014, movement from soybean to alfalfa was at the same level observed within crops, but higher than movement from alfalfa to soybean (Fig 4B). Bi-directional Malaise trap samples showed a similar pattern, higher captures of lady beetles moving from soybean to alfalfa, although it was significant only in the second year of study (mean beetles / bottle / day ± SE; 2013: alfalfa to soybean: 0.50 ± 0.34, soybean to alfalfa: 1.67 ± 0.58, paired t = 1.40, df = 5, *p* = 0.22; 2014: alfalfa to soybean: 0, soybean to alfalfa: 1.05 ± 0.32, one sample t = 3.28, df = 19, *p* < 0.05). Displacement distance and speed of displacement followed similar patterns, being generally higher from soybean to alfalfa in both experiments (S1 Appendix).

No aphids were observed in the soybean fields studied. In contrast, high number of aphids were found in alfalfa, with higher populations in 2014 (210.33 ± 61.65 aphids / 25 sweeps) than in 2013 (58.33 ± 9.41 aphids / 25 sweeps; *t* = - 2.43, df = 10, *p* < 0.05). Pea aphids dominated the assemblage of aphids in alfalfa (97.7%), followed by spotted alfalfa aphids (2.3%). Low numbers of aphidophagous predators were found using sweep-net sampling, but predator abundance was higher in alfalfa than in soybean (2013: alfalfa = 2.33 ± 0.71 individuals / 25 sweeps, soybean = 0.33 ± 0.33, *F*_1,10_ = 6.43, *p* = 0.03; 2014: alfalfa = 3.50 ± 1.09, soybean = 0.33 ± 0.21 individuals / 25 sweeps, *F*_1,10_ = 8.17, *p* = 0.02).

## Discussion

Our study contributes the first empirical evidence from North America that suggests that movement of predators into crops depends on both predator identity and the type of adjacent habitat, confirming patterns found in other regions [4–7, 24]. Directionality in control traps (located 100 m from the border) of soybean fields did not differ for all predators combined or for most predator groups separately, suggesting that the difference in directionality observed in field borders was due to the influence of adjacent habitats and not to other factors (such as wind direction, trap orientation, etc.). Interestingly, the quantities of all groups of predators captured in the interior of soybean fields were similar to those captured in field borders, indicating that foraging predator activities continue from the field border to near the centre of the fields (at least at the scale of 100 m from the field border).

Generalist predators provided strong suppression of soybean aphids, confirming previous result in other regions of North America [12, 40], including in Manitoba [23]. We demonstrate with experimental field manipulations that exposure to aerial predators alone resulted in the same level of aphid suppression as exposure to both aerial and epigeal predators, supporting previous findings that suggested that aerial predators are responsible for soybean aphid suppression in North America [16]. Similarly, studies in cereal aphids suggest that aerial predators are sometimes more important mortality factors than epigeal predators (e.g. [31], but see [41]). This finding further supports the need to study predator movement across field borders, as aerial natural enemies are the group most likely to spillover from extra-field habitats [42] and provides empirical support for the hypothesis that low numbers of aerial predators from adjacent fields can suppress colonizing populations of pests in crops [20, 28].

Two groups of predators showed similar patterns of movement relative to soybean fields, aphidophagous hover flies (the dominant taxa, with 89% of total captures in Malaise traps) and green lacewings (0.6%), with high emigration to canola (flowering at the time of our study) and high immigration from woodland (significant only for hover flies). A previous study conducted in Australia showed that higher number of hover flies moved from native vegetation to adjacent barley and wheat than vice versa [6], suggesting that woodland may be a good source of hover flies in the agricultural landscape. Lacewings are considered nomadic in field crops, moving in downwind direction to new habitats every couple of days [43]. Lacewings and hover flies search for flowering plants to obtain nectar and pollen to fulfill their nutritional requirements and can facilitate pollination (e.g. [44,45]). The hover fly, *T*. *marginatus*, was the numerically dominant aphidophagous species in our study (85% of total captures in Malaises traps), confirming previous findings in other regions of North America [46,47]. This species acts both as an aphid predator [48] and a pollinator in soybeans [49]. Several aphid species are reported in canola, including the cabbage aphid (*Brevicoryne brassicae* (Linnaeus), the green peach aphid *Myzus persicae* (Sulzer), and the turnip aphid (*Lipaphis erysimi* (Kaltenbach) (Hemiptera: Aphididae) [50], suggesting that hover flies and green lacewings may also provide pest control services to canola. The low number of aphids in our focal soybean fields, and diminishing nectar resources in soybean fields at the end of the flowering period, could explain the patterns of hover fly and lacewing movements towards canola observed in our study. Despite their relatively low number, movement of green lacewing is one of the best explanatory variables for the levels of soybean aphid suppression observed in our study area [23]. Future studies should determine the season-long pattern of movement of hoverflies and green lacewings, particularly at non-flowering periods of canola and soybean, to determine their overall contribution to pollination and pest control services in various crops.

Overall lady beetle movement was higher in fields bordering wheat and alfalfa compared to canola and woodland habitats, in the year with high lady beetle abundance. Macfadyen and Muller [5] observed coleopteran and neuropteran predators moving more frequently from cereal fields (wheat / barley) to canola fields late in the season, suggesting that these predators are frequently associated with cereals early in the season. In Manitoba, wheat fields support several species of lady beetles and aphids, including the English grain aphid, *Sitobion (Macrosiphum) avenae* Fabricius, the bird cherry-oat aphid, *Rhopalosiphum padi* (Linnaeus), and the greenbug, *Schizaphis graminum* (Rondani) [51, 52]. Similarly, several lady beetle and aphid species occur in alfalfa, including pea aphids and spotted alfalfa aphids [29]. Our sticky trap sampling in the cage manipulation study suggests that lady beetles are the dominant aerial predator group in alfalfa. We observed similar levels of soybean aphid suppression in both soybean and alfalfa crops, suggesting that the predator assemblage in alfalfa can suppress aphids on soybean plants. Moreover, the levels of suppression of soybean aphid were similar to those observed on pea aphids on alfalfa plants. Schmidt, O'Neal [53] found soybean grown with an alfalfa living mulch enhances predator diversity and abundance, and increases the suppression of soybean aphids compared to a soybean monoculture. These results suggest that aerial predators in wheat and alfalfa can spillover to neighboring soybean fields and suppress soybean aphid populations.

The results of the mark-release-recapture experiments also suggest that alfalfa is a suitable habitat for lady beetles, with individuals dispersing in greater numbers from soybean to alfalfa. Prevailing wind direction does not explain the patterns of movement observed as wind blew from alfalfa to soybean in both years [33]. A potential explanation for the net movement to alfalfa could be the abundance of pea aphids and spotted-alfalfa aphids in alfalfa, and the absence of aphids in soybean. Lady beetles use “resource mapping” and leave crops to evaluate the quality of the surrounding habitats [54]. Cardinale, Weis [55] observed that after arriving to a habitat, lady beetles decide to stay or leave based on the availability of prey and signals from conspecific larvae. Ives [8] used mark-release-recapture methods to study the abundance and movement of lady beetles between alfalfa and oat plots in British Columbia, Canada. He showed that the two lady beetle species studied move between crops, but *C*. *trifasciata* prefers alfalfa and *C*. *californica* prefers oat. Movements of both species were affected by aphid density in the plots and by temperature. In another mark-release-recapture study, van der Werf, Evans [9] found that *C*. *septempunctata*, moved greater distances and stayed shorter times when aphids were not abundant in alfalfa, even in sugar-sprayed plots. Our findings are consistent with a resource mapping strategy for *C*. *septempunctata* and suggest that alfalfa may supply *C*. *septempunctata* to adjacent soybean fields with aphid infestations.

Studies that quantified agricultural landscape complexity have suggested that woodlands are an important sources of predators to crops (reviewed in [56]). Previous studies showed that increasing proximity to and amount of wooded areas in the landscape increase predator richness and abundance in crops, including soybean [57, 58]. Lady beetle abundance is higher in soybean fields located in complex landscapes associated with more forests and grasslands areas [20]. Despite these patterns, few studies measured the movement of predators directly in relation to woodlands. We found that hover flies, green lacewings, minute pirate bugs, lady beetles, and brown lacewings showed patterns of higher movement from woodland to soybean, than vice versa, although it was significant for the first predator group only, probably due to the low captures by bi-directional Malaise traps observed in the other groups. A study using bi-directional Malaise traps in Córdoba province, Argentina suggested that coleopteran predators move from forest to soybean in greater numbers than vice versa, and movement of predators decreases with senescence of soybean [24]. Macfadyen, Hopkinson [6] found that lady beetles, adult hover flies and brown lacewings moved in greater numbers and more often from native vegetation to adjacent crop fields (i.e. barley and wheat) in New South Wales and Queensland, Australia. In contrast, Macfadyen and Muller [5] found no differences between immigration and emigration of hover flies and lady beetles between native perennial vegetation and canola in New South Wales, Australia. Our results provide further empirical evidence that woodlands function as a source of predators to crops, but additional studies at different times of the season are needed to fully understand the role of woodlands as potential sources of aphidophagous predators to crops.

Patterns of predator movement into crops are crucial to understand pest suppression levels [2, 28]. Our study demonstrates that the directionality of predator movement in soybean borders is significantly affected by the identity of adjacent habitats and differs by predator group. We also demonstrate that aerial predators easily move between neighboring habitats and can suppress soybean aphids, even without the additional contribution of epigeal predators to aphid suppression. Future studies should investigate how crop phenology influences the seasonal pattern of movement of predators, particularly due to the fluctuation of prey and other resources (e.g. [2, 24]). Farmers, policy makers and stakeholders could incorporate this knowledge to determine an ideal configuration of crop fields to enhance natural biological control of pests via increasing the movement of natural enemies into particular crops at critical times during the growing season.

## Supporting information

S1 Fig Captures of aphidophagous predators showing movement trends between soybean and adjacent habitats. (PDF)

S1 Appendix Displacement distance, speed of displacement and additional tests conducted for the mark-release-recapture study. (PDF)

S1 Datasets 1) bidirectional malaise trap predator captures, 2) soybean aphid counts in the exclusion cage experiment, 3) pea aphid counts in the exclusion cage experiment, 4) predator sticky-trap counts in experimental fields, 5) aphid and predator sweep-net counts in experimental fields, 6) counts of C. septempunctata in the mark-release-recapture experiments, 7) distance travelled by C. septempunctata recaptured in the mark-release-recapture experiments, and 8) counts of C. septempunctata in bidirectional Malaise traps in the mark-release-recapture experiments.
(XLSX)
