# Supplementary material for: Adjacent habitat type affects the movement of predators suppressing soybean aphids
Source: PLoS One. 2019 Jun 18;14(6):e0218522. doi: 10.1371/journal.pone.0218522 (PMC6581284; doi:10.1371/journal.pone.0218522)
Supplement: S1 Fig — (PDF) [file pone.0218522.s001.pdf]

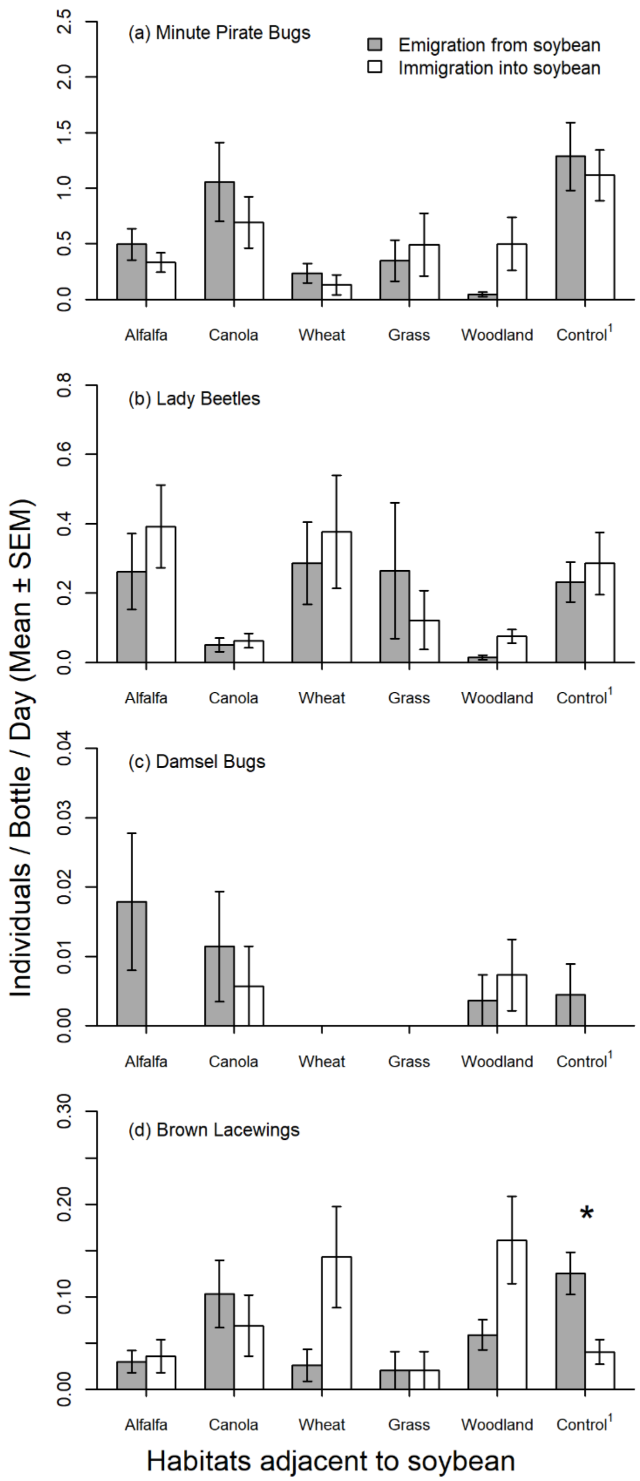

S1 Fig. Captures of aphidophagous predators showing movement trends between soybean and adjacent habitats. Average daily emigration and immigration of (a) minute pirate bugs, (b) lady beetles, (c) damsel bugs, and (d) brown lacewings between soybean and different adjacent

habitats, combining two years of sampling (7 weeks in total). Sampling consisted of bi-directional Malaise traps established on five adjacent habitats: soybean-alfalfa (n= 24 bottles), soybean-canola (n= 25), soybean-grass (n=7), soybean-woodland (n=39), soybean-wheat (n=11) and control (soybean fields, 100 m from the field border, n=32). Significant differences (p < 0.05) between emigration and immigration are indicated by *. [1] Control bi-directional Malaise traps setup in a subset of fields in 2014 (n=8 fields).
