# Supplementary material for: Adjacent habitat type affects the movement of predators suppressing soybean aphids
Source: PLoS One. 2019 Jun 18;14(6):e0218522. doi: 10.1371/journal.pone.0218522 (PMC6581284; doi:10.1371/journal.pone.0218522)
Supplement: S1 Appendix — (PDF) [file pone.0218522.s002.pdf]

S1 Appendix. Displacement distance, speed of displacement and additional tests conducted for the mark-release-recapture study.

Methods

The displacement distance by recaptured beetles was calculated as a straight line between the release and the recapture points. Travel speed was calculated as displacement distance / time. Due to the changes in the sampling design that resulted in variable maximum travel displacement distances monitored within and between fields, separate analyses were conducted per year. Plant height, vegetative and reproductive stages were recorded using 30 randomly selected plants each year. Quadrat sampling (1 m^2 quadrats; 30 quadrats / field) was carried out to measure the percentage of vegetation cover. Plant counts and quadrat sampling were conducted outside the mark-release-recapture sampling area to avoid disturbance. Either paired t-tests or one sample t-tests (when there were no captures in one treatment) were used to compare the directionality of lady beetles captured in bi-directional Malaise trap samples. Due to the different maximum distance at which lady beetles were sampled within and between crops, separate analyses were conducted for displacement distances and speed results, using either two sample t-tests, Wilcoxon rank sum test with continuity correction or one sample t-tests. One-way ANOVA was used to compare plant height and percentage of vegetation cover between alfalfa and soybean within and between years. For all parametric tests, normality of the data and homogeneity of variance were visually checked using normal Q-Q plots and heteroscedasticity plots (i.e. fitted values vs. residuals).

Results

The greatest displacement distances recorded from recaptured marked lady beetles were 33 m after 4 h and 52.4 m after 28 h in 2013 and 2014 respectively, both from soybean to alfalfa. The

maximum displacement distances recorded within alfalfa were 12 m after 4 h and 24.3 m after 32 h, and within soybean 8.5 m and 18.4 m in 2013 and 2014 respectively. In 2013, mean displacement of ladybeetles was higher within alfalfa than within soybean (two sample t = 2.54, df = 31, $p = 0.02$; Fig S2a). The same trend was observed for displacement distances in 2014, but it was not significant (two sample $t = 1.77$, df = 10, $p = 0.11$; Fig S2b). Longer displacement distances were observed from soybean to alfalfa than vice versa in both years (2013: one sample $t = 9.08$, df = 2, $p = 0.01$; 2014: two sample $t = -3.71$, df = 11, $p = 0.0003$; Fig S2a and b).

The highest speeds recorded from recaptured marked lady beetles were 8.25 mh^{-1} (2013) and 18.34 mh^{-1} (2014), both for beetles that moved from soybean to alfalfa. In 2014, lady beetles moved faster from soybean to alfalfa than vice versa (Wilcoxon statistic = 0, $p = 0.03$; Fig S2d). The same trend was observed for speeds recorded in 2013, but it was not significant (one sample $t = 3.63$, df = 2, $p = 0.07$; Fig S2c). Higher speeds were recorded within alfalfa than in soybean in 2013 (two sample t = 2.20, df = 31, $p = 0.04$; Fig S2c). The same pattern was observed for speeds within crops in 2014, but it was not significant (Wilcoxon statistic =45, $p = 0.35$; Fig S2d).

Plant heights of both soybean and alfalfa were greater in 2014 than 2013 (soybean: 25.50 ± 0.24 cm in 2013, 43.64 ± 0.89 cm in 2014, $F_{1,58} = 386.1$, $p < 0.001$; alfalfa: 24.97 ± 0.13 cm in 2013, 41.30 ± 1.36 cm in 2014, $F_{1,58} = 142.4$, $p < 0.001$). However, similar plant heights were recorded between crops within the same year (2013: $F_{1,58} = 3.67$, $p = 0.06$; 2014: $F_{1,58} = 2.06$, $p = 0.16$). The percentage of vegetation cover in soybean was higher than in alfalfa in both years (2013: soybean 61.83 ± 2.76 %, alfalfa 42.33 ± 2.27 %, $F_{1,58} = 29.77$, $p < 0.001$; 2014: soybean 65.50 ± 2.88 %, alfalfa 39.10 ± 3.03 %, $F_{1,58} = 39.83$, $p < 0.001$), but similar vegetation covers

were recorded in the same crop type between years (soybean: $F_{1,58} = 0.85$, $p = 0.40$; alfalfa: $F_{1,58} = 0.78$, $p = 0.36$).

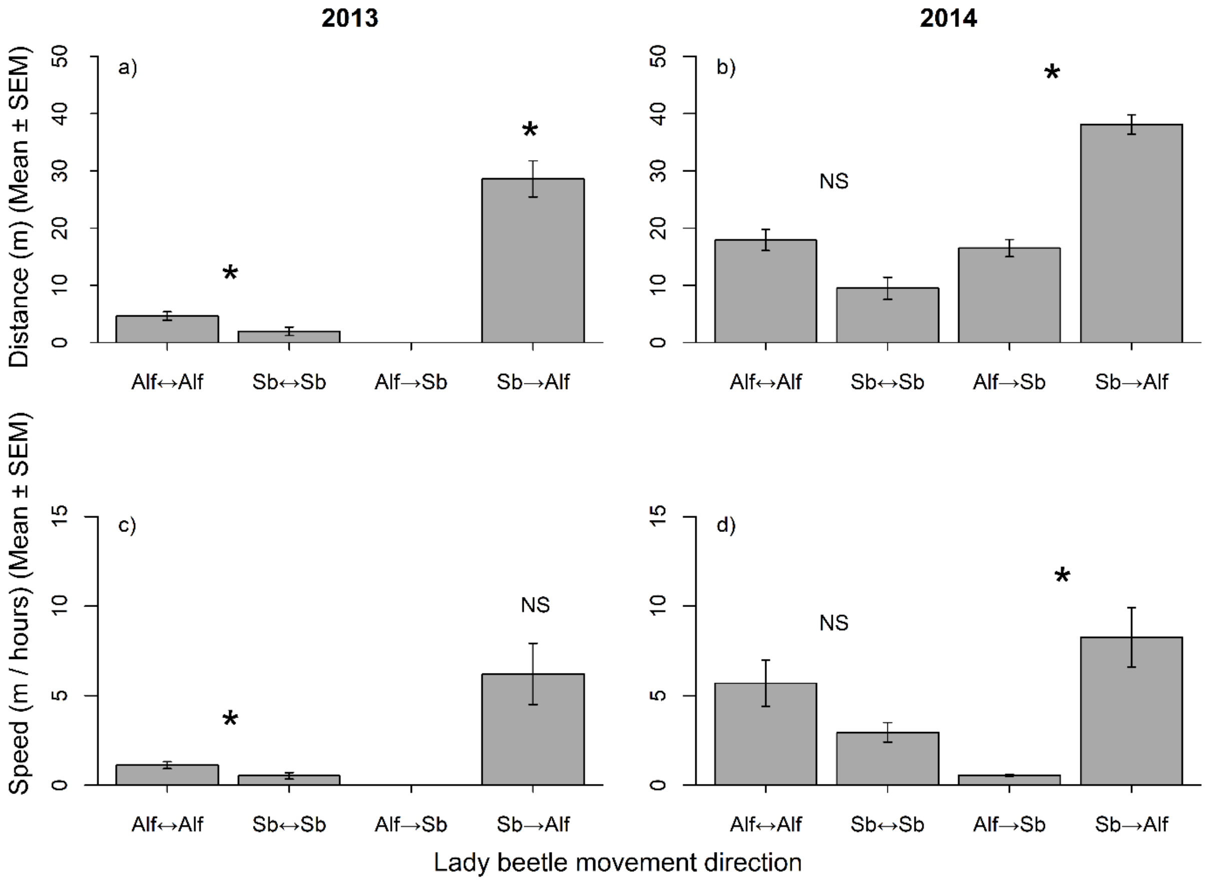

Fig S2. Displacement distances (a and b) and speeds (c and d) of marked lady beetles, *C. septempunctata,* within and between soybean and alfalfa fields in 2013 and 2014. Significance of differences between displacement distances (a and b) and speed of marked beetles (c and d) within and between fields in each year was determined by a two-sample t-test or Wilcoxon rank sum test with continuity correction. When one of the treatments was zero, a one sample t-test with H_o Mean = 0 was used. * indicates $p < 0.05$; NS = not significant. Movement directions: Alf↔Alf: alfalfa to alfalfa, Sb↔Sb: soybean to soybean, Alf→Sb: alfalfa to soybean, Sb→Alf: soybean to alfalfa.
